# Associations of Affect, Action Readiness, and Sexual Functioning

**DOI:** 10.1016/j.esxm.2020.06.005

**Published:** 2020-07-05

**Authors:** Marcus J.M.J. Henckens, Peter de Vries, Erick Janssen, Thomas De Sutter, Anja J.H.C. van den Hout, Susan A.H. van Hooren, Jacques J.D.M. van Lankveld

**Affiliations:** 1 Faculty of Psychology, Open University of the Netherlands, Heerlen, The Netherlands; 2 Department of Urology, Zuyderland MC, Heerlen, The Netherlands; 3 Institute for Family and Sexuality Studies, Department of Neurosciences, University of Leuven, Belgium; 4 Department of Urology, Ziekenhuis Oost-Limburg, Genk, Belgium; 5 Clinical and Medical Psychology, Zuyderland MC, Heerlen, The Netherlands; 6 KenVaK, Research Centre for the Arts Therapies, Heerlen, The Netherlands; 7 Zuyd University of Applied Sciences, Faculty of Healthcare, Heerlen, The Netherlands; 8 Open University, Faculty of Psychology, Heerlen, The Netherlands

**Keywords:** Action Readiness, Affect, Affective Polarity, Trait and State Measurements, Male Sexual Functioning

## Abstract

**Introduction:**

Emotions are theorized to contain the components of affect and action readiness. Affect guides behavior by causing an approach or withdrawal orientation. Action readiness is the individual's degree of willingness to interact with the environment. Emotions contribute to changes in behavior and physiological responses.

**Aim:**

The present study applied these notions to sexuality and examined the associations between affect, action readiness, and sexual functioning.

**Methods:**

Participants were male patients with urologic condition (N = 70) with and without sexual problems.

**Main Outcome Measure:**

Affect and action readiness were jointly assessed using the latent factor of affective polarity of the Positive and Negative Affect Schedule. Trait affective polarity was assessed questioning generally experienced feelings. State affective polarity was assessed after exposure to an erotic stimulus and questioning momentaneously experienced feelings. Sexual functioning was assessed using the International Index of Erectile Functioning questionnaire.

**Results:**

A significant increase of approach-oriented action readiness was found after erotic stimulation, relative to trait levels. In addition, significant associations were found between state approach-oriented action readiness and various aspects of sexual functioning. Interventions based on principles of positive psychology might be developed to reinforce action readiness in men with erectile dysfunction. The strength of the current research concerns the introduction of action readiness as a potential psychological factor implied in sexual functioning. Limitations pertain to the use of the algorithm used to calculate state approach-oriented action readiness and the use of the current sample of patients with urological conditions, limiting generalizability of findings.

**Conclusion:**

Action readiness was found to correlate positively with all aspects of sexual functioning. Further research into the role of action readiness in sexuality is recommended.

**Henckens MJMJ, de Vries P, Janssen E, et al. Associations of Affect, Action Readiness, and Sexual Functioning. Sex Med 2020;8:691–698.**

## Introduction

The present study investigates the associations of affect and action readiness with different aspects of male sexual functioning. In the appraisal theory of emotion by Frijda,^1^, ^2^, ^3^ it is postulated that expectations of reward or punishment elicit positive affect (PA) or negative affect (NA), respectively.^1^^,^^4^ In addition to affect, the same expectations also elicit action readiness,^5^ defined as the degree of willingness to interact with the environment.^4^ Together, affect and action readiness can be seen as constituting a factor that we call approach- (or avoidance-) oriented action readiness.^5^ It represents the strength (the action readiness component) of the tendency to approach or avoid (the affective component) emotionally relevant stimuli.

Emotions can be distinguished by their specific patterns of approach vs withdrawal orientation and action readiness.^1^^,^^6^ Sexual arousal shares several characteristics with other emotions and is a potent motivator for approach behavior.^7^ A further distinction to be made involves the difference between state and trait emotions.^8^ State emotions are transient responses to instantaneous stimuli.^9^ In contrast, trait emotions are relatively stable and correlate with personality traits of neuroticism and extraversion.^10^^,^^11^ Investigating state and trait emotions, specifically focusing on approach-oriented action readiness, might therefore contribute to the understanding of the determinants of sexual functioning and dysfunction.

Research exclusively focusing on the role of momentary action readiness in sexual functioning is scarce. Both et al^12^ investigated the effect on sexual arousal of action readiness in the form of motor preparation, operationalized as the strength of the Achilles tendon reflex in response to erotic stimuli. Reflex strength was higher in response to more intense sexual stimuli. Although this study provides the most direct evidence of involvement of the action readiness component of emotion, the causal direction that was investigated, ie, the effect of sexual stimulation on action readiness, does not answer the research question of the present study how both state and trait action readiness are associated with sexual functioning.

More research has been conducted on affective states and traits as determinants of sexual functioning. However, these studies have yielded divergent findings, and none of them included a measure of action readiness.^13^^,^^14^ As for affective state, and its association with sexual functioning, as assessed using sexual response measures, Peterson and Janssen^15^ found that the simultaneous experience of high PA and high NA was associated with higher levels of subjective sexual arousal and sexual desire in men, whereas in other studies, positive and negative emotional states were both found to separately facilitate sexual functioning.^16^^,^^17^ In contrast, a study of Carvalho et al^18^ did not reveal effects of emotional state induction on attentional processes during visual erotic stimulation. Yet, men with sexual dysfunction (SD) reported stronger negative emotional states in sexual situations than sexually functional men.^19^^,^^20^ In sum, findings regarding the associations between affective states and sexual functioning are mixed.

Studies on the association between emotional traits and sexuality have included erotophobia-erotophilia,^21^ a dimension of affective and evaluative responses to sex. A person's position on this dimension broadly predicts his or her tendency to approach or avoid sexual situations. In one of the few experimental studies on erotophobia-erotophilia, a priming paradigm was used with backward masking of the primes.^22^ In this study, erotophobic and erotophilic respondents did not differ in the recognition of sexual target stimuli. However, erotophilic respondents demonstrated automatic associations between sexual priming stimuli and (non-sexual) positively valenced targets. In contrast, erotophobic individuals classified negatively valenced targets faster regardless of whether primes were sexual or neutral.

Another line of research suggests that the impact of NA on sexual functioning is moderated by other individual traits, including the propensity for sexual excitation and inhibition.^23^^,^^24^ This work starts from the premise that individual variability in sexual responsiveness is predicted by an individual's propensity for the assumed independent processes of sexual excitation and sexual inhibition proneness.^25^ It has been found that NA, although it generally had a negative effect on sexual motivation, can increase sexual motivation in men with a low propensity for sexual inhibition.^23^ In general, it seems justified to conclude that the connection between PA and NA and sexual functioning is complex and not yet well understood. Against this backdrop, including action readiness and the interactions of affect and action readiness in the study of sexual functioning may increase our understanding of underlying processes and mechanisms. In particular, the distinction between state and trait aspects of action readiness may have additional value.

### Measuring Action Readiness Using the Positive and Negative Affect Schedule

For measuring approach-oriented action readiness, we used the Positive and Negative Affect Schedule (PANAS).^8^ The PANAS is based on the “circumplex” model of emotions^26^^,^^27^ and relies on the notion that high affective states are accompanied by behavioral tendencies.^28^ The PA gradient ranges from feeling leisurely to energetic enthusiasm, whereas the NA gradient ranges from calm, tranquil feelings to anxiety and distress.^27^

An additional latent factor was identified in the PANAS, which was termed affective polarity (AP).^29^, ^30^, ^31^ It reflects the strength of the individual's orientation toward withdrawal vs approach.^29^ It is also seen as reflecting aspects of self-determination in approach behavior,^30^ although this latter view has been disputed.^32^ Using AP, Leue and Beauducel^29^ were able to differentiate between impulse-controlled disordered sex offenders, other sex offenders, and non-offenders. Because of its conceptual overlap with the combination of affect and action readiness, AP was used in the present study as operationalization of approach-oriented action readiness.

In previous research, substantial associations,including sexual desire, sexual arousal and erectile functioning, orgasm, and sexual satisfaction, were found between different aspects of sexual functioning in healthy aging men^33^ and men with prostate cancer.^34^ In the present study, we predicted significant positive associations of state levels of approach-oriented action readiness with these aspects of sexual functioning, also when trait action readiness is controlled for. Furthermore, as we assumed age effects—consistent with previous findings of higher levels of emotional well-being^35^ and lower levels of sexual functioning with increasing age^36^—we decided to control for age in the analyses. The following hypotheses were tested: (i) Exposure to an erotic stimulus will increase state AP, relative to the AP trait level; (ii) State AP correlates positively with all aspects of sexual function; and (iii) State AP correlates positively with all aspects of sexual function, after controlling for age and trait AP. The impact of SD and medication use on the associations of AP with sexual functioning was exploratively examined.

## Method

### Participants

Participants were heterosexual male patients visiting an outpatient urology clinic at Zuyderland hospital in Heerlen, the Netherlands, for urological problems, sexual complaints, or both. Men with urological conditions without sexual problems were included to ensure sufficient variability in sexual functioning within an otherwise homogeneous sample. In addition, limiting the sample to sexually functional and healthy subjects might have obscured the existence of differences with regard to associations between action readiness and aspects of sexual functioning. Exclusion criteria were high level of psychopathology, insufficient mastery of the Dutch language, and severe or acute somatic diseases, including oncologic conditions. Power analysis^37^ yielded a sufficient number of participants of 82 for partial correlation analysis (2-tailed, medium effect size (0.3), α = 0.05, power = 0.80).

### Instruments

#### Demographic, Medical, and Sexual Health–Related Information

Demographic and self-reported sexual health information was collected. Medical information, including medication use possibly affecting sexual functioning, was obtained from the urologist.

#### Sexual Functioning

Level of sexual functioning was assessed using the 15-item International Index of Erectile Functioning (IIEF) questionnaire,^38^ with subscales for erectile function, orgasmic function, sexual desire, intercourse satisfaction, and overall sexual satisfaction. Higher scores represent better sexual functioning. Information on the reliability of all instruments in the present sample is presented in Table 1.

**Table 1:** Reliability coefficients

Scale	N	Cronbach's α [95% CI]	ω (total) [95% CI]	GLB
HADS	83	.82 [.77, .88]	.83 [.78, .88]	.90
Trait PANAS-PA	70	.88 [.84, .92]	.68 [.84, .92]	.93
Trait PANAS-NA	70	.83 [.70, .86]	.83 [.70, .85]	.92
Trait PANAS-AP	70	.86 [.69, .85]	.79 [.72, .86]	.88
State PANAS-PA	70	.94 [.92, .96]	.94 [.92, .96]	.97
State PANAS-NA	70	.82 [.76, .89]	.81 [.74, .88]	.92
State PANAS-AP	70	.75 [.67, .83]	.75 [.66, .84]	.92
IIEF sum score	70	.97 [.96, .98]	.97 [.97, .98]	.98
IIEF-EF	70	.97 [.96, .98]	.97 [.96, .98]	.97
IIEF-OF	70	.95∗	(*r* = .90)∗	
IIEF-SD	70	.88∗	(*r* = .79)∗	
IIEF-IS	70	.93 [.90, .96]	.95 [.93, .97]	.95
IIEF-OS	70	.88∗	(*r* = .79)∗	

EF = erectile function; GLB =Greatest Lower Bound; HADS = Hospital Anxiety and Depression Scale; IIEF = International Index of Erectile Function; IS = intercourse satisfaction; NA = negative affect; OF = orgasmic function; OS = overall sexual satisfaction; PA = positive affect; PANAS = Positive and Negative Affect Schedule; SD = sexual desire.

∗ Not enough items to calculate the confidence interval.

#### Psychopathology

Anxiety and depression were measured using the 14-item Dutch version of the Hospital Anxiety and Depression Scale questionnaire.^39^ In the Dutch version of the Hospital Anxiety and Depression Scale, the sum score is more sensitive than its subscales,^40^ and it is suitable as a screening instrument.^41^ Sum scores >15 indicate higher levels of psychopathology.

#### PA, NA, and AP

The PANAS was used to measure PA, NA, and AP. The PA and NA subscales both contain 10 items,^8^ with scores ranging from 10 to 50. Higher scores indicate higher affective states.^28^ This study used the Flemish version of the PANAS because of its better internal consistency of the PA subscale compared with the Dutch version.^42^^,^^43^ AP scores were calculated by subtracting scores on selected NA items from those on selected^a^ PA items,^29^ with scores ranging from −33 to 3. As this allows for a partly shared variance of AP scores with PA and NA, only AP scores were used for hypothesis testing. Higher AP scores indicate higher level of approach-oriented action readiness, whereas low scores indicate stronger avoidance-oriented action readiness. Trait AP measurement took place at the beginning of the test session, by asking participants about feelings they generally experience. At the end of the session, state AP was measured by asking for momentaneous feelings, immediately after a 20-second presentation of an on-screen color image (500 × 300 pixels; screen resolution 1920 × 1080) composed of 4 erotic pictures that were previously used in laboratory research to induce sexual arousal.^44^ The pictures depicted heterosexual penile-vaginal penetration. Induction of sex-related emotions using visual presentation of still erotic pictures for periods as brief as 8 seconds has been found efficacious in previous research.^45^^,^^46^

#### Procedure

Subsequent patients visiting the urology outpatient clinic were invited to participate in this study. Interested patients received a leaflet with information about the study and its procedures. Patients who expressed their interest received a written summary of their urological status including any diagnosis of SD from their doctor. A research session was scheduled in which the test procedure was further explained by the researcher (M.H.). After signing an informed consent form, participants handed over the doctor's summary to the researcher and completed the questionnaires on a computer, as described previously, while ensuring sufficient privacy. The study was approved by the hospital's ethical review board.

### Statistical Analysis

IBM SPSS 24 was used for hypothesis testing. Scale reliability, including Cronbach's alpha, McDonald's omega, and Greatest Lower Bound,^47^ was calculated using R, version 3.3.3. The effect of mood induction on AP was tested using repeated-measures ANOVA with trait level of AP as first and state level of AP after erotic induction as second measurement. Effects were controlled for age in these analyses. Associations of state AP with aspects of sexual functioning were calculated using partial correlation analysis (2-tailed), owing to collinearity between age and AP. The analyses were repeated while controlling for both age and trait AP. A significance threshold of *P* < .01 was applied to reduce the risk of inflated type-1 error because of multiple testing. Effect sizes were interpreted following Cohen's^48^ guidelines.

## Results

### Preliminary Analyses

70 patients participated (M_age_ = 55.7 years, *SD* = 14.9). 36 (51.4%) had an SD diagnosis, 8 men (11.4%) reported experiencing sexual problems but had no SD diagnosis, and 26 participants (37.2%) did not report sexual problems nor had an SD diagnosis (Table 2). Sexual problems were low sexual desire (14%), erectile problems (57%), low subjective sexual arousal (13%), delayed orgasm (31%), premature ejaculation (29%), and genital pain (6%). Medical conditions in participants with diagnosed SD were lower urinary tract symptoms (15%), prostate disease (15%), hematuria (4%), kidney stones (12%), scrotum/testes complaints (8%), narrow foreskin (4%), penile deformity (4%), genital skin problem (4%), and 42% requested sterilization. Medical conditions in participants without diagnosed SD were lower urinary tract symptoms (48%), prostate disease (25%), kidney stones (5%), scrotum/testes complaints (7%), endocrine condition (2%), and 5% requested sterilization. Medication use was recorded as erection-enhancing (eg, phosphodiesterase type 5 inhibitor; 12%), potentially erection-impairing (eg, selective serotonin reuptake inhibitors; 16%), other (32%), and no (40%) medication.

**Table 2:** Demographic and sexual dysfunction characteristics (n = 70)

Characteristic	Total
Age	55.1 (14.9)
Highest achieved education level	
Elementary	3 (4.3%)
Lower secondary	12 (17.1%)
Higher secondary	34 (48.6%)
College	8 (11.4%)
Professional	11 (15.7%)
University	2 (2.9%)
Relationship	
Married/living together	53 (75.7%)
Partnered/not living together	6 (8.6%)
No relationship	11 (15.7%)
Relationship duration in years	27.6 (16.3)
Children (% yes)	58 (82.9%)
Sexual dysfunction (SD) diagnosed	36 (51.4%)
Self-reported sexual problems (no SD diagnosis)	8 (11.4%)
No sexual problem (no SD diagnosis)	26 (37.2%)

To examine the associations of age with the study variables, multiple linear regression was performed with age as a criterion variable. Trait AP, state AP, and IIEF subscale scores were entered as predictor variables. A significant regression model was found (*F*(7,62) = 5.84, *P* < .001). Higher age was associated with lower sexual desire, lower erectile and orgasmic function, and lower satisfaction with intercourse but was not associated with trait AP, state AP, or with overall sexual satisfaction. Age was used as a covariate in further analyses.

To explore the effects of medication use on the study variables, multivariate analysis of covariance was performed with medication use (no, erection-enhancing, erection-impairing, and other medication) as grouping factor. Age was entered as a covariate, and trait AP, state AP, and IIEF subscale scores were entered as dependent variables. The multivariate effect of medication use on the dependent variables was not significant. Medication use was omitted in further hypothesis testing.

After multivariate analysis of covariance with SD (diagnosed or self-reported vs no SD) as grouping factor, age as covariate, and trait AP, state AP, and IIEF subscale scores as dependent variables, a significant multivariate effect of SD was found (*F*[7,61] = 14.54, *P* < .001). SD was associated with lower state AP, lower sexual desire, lower erectile and orgasmic function, lower satisfaction with intercourse, and lower overall sexual satisfaction but was not associated with trait AP. When medication use was added as covariate to this analysis, the same pattern of results was found.

Repeated-measure ANOVA was performed to test the effects of sexual arousal induction on state AP, compared with trait AP. A medium-sized within-subjects difference was found, *F*(1,69) = 41.49, *p*(1-tailed) < 0.001, partial η^2^ = 0.38; M_traitAP_ = -6.29 (*SD* = 4.84); M_stateAP_ = -2.24 (*SD* = 4.03), confirming the effect of erotic stimulation on state AP.

Trait and state AP correlated with a medium effect size (*r* = 0.35). Partial correlations, correcting for age and trait AP, were computed between state AP and IIEF subscale scores (see Table 3). Significant correlations of medium effect size were found between state AP and sexual desire, erectile function, orgasmic function, intercourse satisfaction, and overall satisfaction. Higher levels of sexual functioning were associated with higher state AP, independent of age and trait AP.

**Table 3:** Partial correlation coefficients of age, trait AP, state AP, and sexual functioning (*n* = 70)

	Age	Trait AP	State AP‡	State AP§	IIEF-SD	IIEF-EF	IIEF-OF	IIEF-IS
Trait AP	.01							
State AP	.12	.35∗						
IIEF-SD	−.28	.08	.43†	.42†				
IIEF-EF	−.38∗	.10	.37∗	.36∗	.41†			
IIEF-OF	−.47∗	.10	.40†	.38†	.50†	.79†		
IIEF-IS	−.32∗	.27	.43†	.40†	.47†	.86†	.77†	
IIEF-OS	−.04	.19	.33∗	.30∗	.40†	.60†	.67†	.75†

EF = Erectile Function; IEF = International Index of Erectile Function; IS = Intercourse Satisfaction; OF = Orgasmic Function; OS = Overall Sexual Satisfaction; PA = positive affect; SD = Sexual Desire.

∗ *P* < .01.

† *P* < .001.

‡ Corrected for age.

§ Corrected for age and trait AP

## Discussion

Central to our study aim, state approach-oriented action readiness was found to correlate positively with all aspects of sexual functioning. After controlling for age and trait AP, all correlations remained significant All hypotheses were supported. Although the cross-sectional study design does not allow causal inferences, the finding of increased AP after erotic stimulation provides preliminary support for the emotion theory of Frijda^1^ postulating that sex-related stimuli produce pleasure, when reward is expected.^1^^,^^2^^,^^5^ Sexual function may be better understood and variability in sexual response and behavior may potentially be predicted more accurately when individual variations in action readiness are taken into account. According to Frijda's^49^ theory, action readiness is an elementary aspect of how individuals interact with the social and physical world. Information processing serves to convert the available action repertoire and associated control processes from action readiness into action tendencies. The latter have a stronger association with behavior than action readiness because they involve concrete representations of possible behavioral actions. Specific sexual behaviors are thus guided by expectations more than by situational characteristics *per se*.

The distinction between trait and state aspects of action readiness is of relevance here. Trait action readiness can be assessed using questionnaire measures, whereas the assessment of state action readiness requires the activation or induction of this emotion component. A previous experimental study revealed that sexual arousal, as induced using erotic stimuli, increased action readiness, indicated by a stronger Achilles tendon reflex.^12^ In addition, reflex strength was positively associated with stimulus intensity. The findings of the present study build on this work and show that state action readiness after the induction of sexual arousal is associated with sexual functioning.

For all aspects of sexual functioning, a positive association with state AP was found, after controlling for age and trait AP. This raises the question if sexual functioning could be improved as a result of therapeutic interventions that increase action readiness. From the perspective of positive psychology,^50^ a beneficial approach could be to increase PA before sexual activity to induce a higher state of general approach-oriented action readiness. Indeed, recent research into effects of mindfulness training suggests better sexual outcomes, partly explained by improved emotion regulation.^51^ Another possible modification of AP might be pursued by organizing positive experiences. Positive sexual encounters presumably enhance action readiness combined with an approach orientation through reinforcement learning.^52^^,^^53^ Future research is needed to see whether improving sexual function by modifying action readiness is feasible, despite the entrenched nature of sex-related affect.

### Limitations

A limitation of this study is that our sample, consisting of patients with urological conditions, is not representative of the general population, constraining the generalizability of the present findings. In addition, a self-selection bias may have played a role,^54^ given observations in previous research that individuals with higher sexual interest and more liberal sexual attitudes tend to be more inclined to participate in sex research.^55^

Another limitation is the lack of 1 or more control conditions for the induction of state sexual arousal. Although after erotic stimulation, state approach-oriented action readiness levels increased relative to trait levels, implicating a stronger approach orientation, the specificity of the effect of that manipulation is not warranted, as it cannot be excluded that other factors were responsible for it, including PA and NA, or non-sexual arousal. Future studies should include different control conditions for the manipulation of state AP, to control for the effects of neutral and non-sexual affective states.

Another possible limitation concerns the algorithm that was used to calculate AP. The items used in the algorithm were based on a German version of the PANAS,^29^ whereas the current study used the Flemish translation of the PANAS.^43^ The effect of language differences on the measurement of AP may, nevertheless, be small because of the language- and culture-independent nature of emotional categories.^56^ Furthermore, the AP measure used in this study was based on a sample comprising both men and women.^29^ It is assumed that PANAS results can be generalized across gender.^30^ However, a validation study of the Dutch translation of the PANAS showed a gender difference regarding NA.^42^

Further research on AP in relation to sexual functioning is recommended, especially regarding the causal relation between state AP and physiological responses. It might shed new light on the importance of the role of emotions in sexual responding.

## Statement of authorship

**Category 1**
(a) Conception and Design
  Marcus J.M.J. Henckens; Jacques J.D.M. van Lankveld
(b) Acquisition of Data
  Marcus J.M.J. Henckens; Peter de Vries; Thomas De Sutter
(c) Analysis and Interpretation of Data
  Marcus J.M.J. Henckens; Jacques J.D.M. van Lankveld

**Category 2**
(a) Drafting the Article
  Marcus J.M.J. Henckens
(b) Revising It for Intellectual Content
  Marcus J.M.J. Henckens; Erick Janssen; Anja J.H.C. van den Hout; Susan A.H. van Hooren

**Category 3**
(a) Final Approval of the Completed Article
  Marcus J.M.J. Henckens; Peter de Vries; Erick Janssen; Thomas De Sutter; Anja J.H.C. van den Hout; Susan A.H. van Hooren; Jacques J.D.M. van Lankveld

## Footnotes

^a^ Scores on items 12 (*alert*) and 16 (*determined*) were added, and scores on items 2 (*distressed*), 6 (*guilty*), 7 (*scared*), 13 (*ashamed*), 15 (*nervous*), 18 (*jittery*) and 20 (*afraid*) where subtracted to calculate AP scores.
